# High-throughput identification of protein functional similarities using a gene-expression-based siRNA screen

**DOI:** 10.1038/s41597-020-0365-2

**Published:** 2020-01-21

**Authors:** Beth K. Neilsen, David L. Kelly, Binita Chakraborty, Hyun Seok Kim, Michael A. White, Robert E. Lewis, Kurt W. Fisher

**Affiliations:** 1 Eppley Institute, Fred & Pamela Buffett Cancer Center, University of Nebraska Medical Center, Omaha, NE 68198 USA; 2 Duke University Medical Center, Pharmacology and Cancer Biology, Durham, NC 27710 USA; 3 Severance Biomedical Science Institute, Yonsei University College of Medicine, Seoul, Korea; 4 Department of Cell Biology, University of Texas Southwestern Medical Center, Dallas, TX 75390 USA; 5 Department of Pathology and Microbiology, University of Nebraska Medical Center, Omaha, NE 68198 USA

**Keywords:** Cancer, Cell biology, Molecular biology

## Abstract

A gene expression-based siRNA screen was used to evaluate functional similarity between genetic perturbations to identify functionally similar proteins. A siRNA library (siGenome library, Dharmacon) consisting of multiple siRNAs per gene that have been pooled in to one well per gene was arrayed in a 384-well format and used to individually target 14,335 proteins for depletion in HCT116 colon cancer cells. For each protein depletion, the gene expression of eight genes was quantified using the multiplexed Affymetrix Quantigene 2.0 assay in technical triplicate. As a proof of concept, six genes (BNIP3, NDRG1, ALDOC, LOXL2, ACSL5, BNIP3L) whose expression pattern reliably reflect the disruption of the molecular scaffold KSR1 were measured upon each protein depletion. The remaining two genes (PPIB and HPRT) are housekeeping genes used for normalization. The gene expression signatures from this screen can be used to estimate the functional similarity between any two proteins and successfully identified functional relationships for specific proteins such as KSR1 and more generalized processes, such as autophagy.

## Abstract (summary)

**Table:** 

Measurement(s)	gene expression • KSR1 pathway regulation
Technology Type(s)	Microarray • RNA interference
Factor Type(s)	target RNA
Sample Characteristic - Organism	Homo sapiens

Machine-accessible metadata file describing the reported data: 10.6084/m9.figshare.11418018

## Background & Summary

In 2004, Stegmaier *et al*. described the successful implementation of gene expression-based high-throughput screening to identify chemical compounds that induce differentiation of acute myeloid leukemia cells^1^. This study demonstrated that a gene expression signature could be used as a proxy for a phenotype of interest to allow effective assessment of the functional effect of a chemical or genetic perturbation without examining, or even knowing, the intermediate steps in the pathway driving the phenotype. Properly framed, this innovative and unbiased approach created the potential for identifying novel therapeutic targets for any disease.

To identify novel targets for cancer therapy and expand our fundamental knowledge regarding which proteins are required for specific cellular processes, a gene expression-based screening approach was developed using an eight mRNA signature and a siRNA library was used to deplete 14,355 proteins one at a time in K-Ras (Entrez gene ID: 3845) mutant colon cancer cell line HCT116 and the resulting signatures were compared to identify functionally similar proteins.

**Fig. 1:**
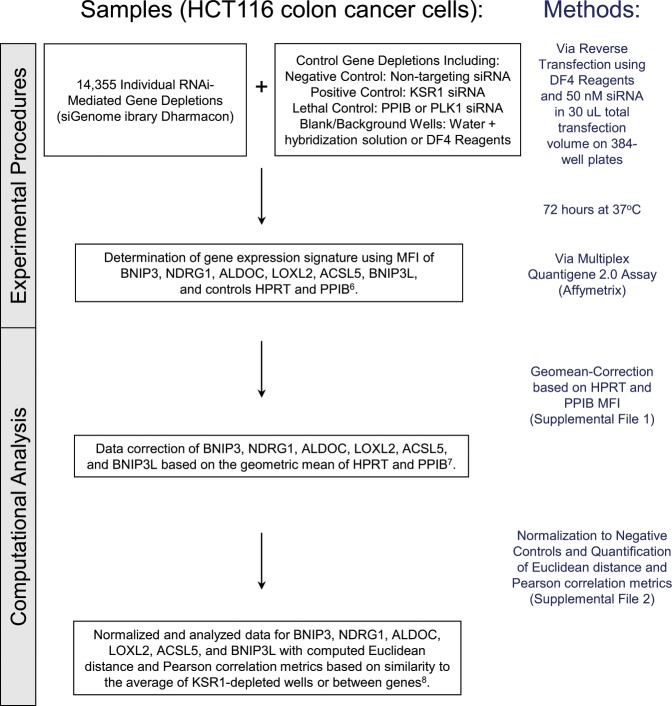
Schematic overview of the experimental procedure and data processing methods. The effect of 14,355 individual gene depletions via siRNA on gene expression of BNIP3, NDRG1, ALDOC, LOXL2, ACSL5, BNIP3L and two control genes HPRT and PPIB was evaluated to determine the functional similarity between KSR1 and each gene screened.

To demonstrate feasibility, a gene-expression signature representing depletion of KSR1 (Entrez gene ID: 8844), a molecular scaffold essential to Ras-driven tumorigenesis^2^, was determined by depleting HCT116 cells of KSR1 using two stably expressed shRNAs (shRNA #1 5′GTGCCAGAAGAGCATGATATT & shRNA #2 5′GCTCTTCAAGAAAGAGGTGAT) and gene expression was compared to parental cells and one non-targeting shRNA (5′ CAACAAGATGAAGAGCACCAA)^3^. Total RNA from these cells was hybridized to Affymetrix Human Genome U133A 2.0 gene chips and six genes (BNIP3 (Entrez gene ID: 664), NDRG1 (Entrez gene ID: 10397), ALDOC (Entrez gene ID: 230), LOXL2 (Entrez gene ID: 4017), ACSL5 (Entrez gene ID: 51703), BNIP3L/NIX (Entrez gene ID: 665)) demonstrating consistent downregulation upon acute KSR1 depletion were chosen for the KSR1-depletion gene expression-based signature. Two additional reporters, cyclophilin B (PPIB - Entrez gene ID: 5479) and hypoxanthine-guanine phosphoribosyltransferase (HPRT - Entrez gene ID: 15452) that did not change with KSR1-depletion were included as controls in an attempt to mitigate confounding variables, such as changes in cell number caused by decreased growth or viability. In contrast to the study performed by Stegmaier *et al*. that measured gene expression using RT-PCR followed by mass spectrometry, the gene expression-based signature in our screen was measured using the Affymetrix Quantigene 2.0 Multiplex assay that allows for multiplexing of samples, which dramatically increases assay throughput. The gene-expression signature for KSR1-depletion was validated using transient siRNA-mediated depletion of KSR1 and all 8 reporter genes were measured using the Quantigene 2.0 Multiplex assay either as a single panel or in a multiplexed format and scaled to 384-well format. A siRNA near genome-scale screen was robotically performed in technical triplicate using a wet reverse transfection. Proteins were individually depleted using multiple pooled siRNAs from the siGenome library (Dharmacon) and gene expression of BNIP3, NDRG1, ALDOC, LOXL2, ACSL5, BNIP3L/NIX, PPIB, and HPRT were measured in a multiplexed fashion (Affymetrix Quantigene 2.0 Multiplex assay) and quantified by reading the median fluorescence intensity of at least 50 beads per gene on a Luminex100 instrument (Fig. 1).

Computational analysis included background subtraction, adjustment of negative numbers, and geometric mean normalization based on PPIB and HPRT. Data was further normalized using negative control wells (wells treated with non-targeting siRNA), log 2 transformed, and the similarity between KSR1 depletion and each individual gene depletion was quantified using Euclidean distance and Pearson correlation similarity metrics (Fig. 1). Additionally, the functional similarly can be assessed with unsupervised clustering methods for any gene of interest.

In summary, this screen generated an 8 mRNA gene-expression signature for the depletion of 14,355 proteins and led to numerous biologically validated but previously unknown functional relationships^4,5^, including novel relationships for KSR1^3^. In the text, we discuss the methodology use to conduct the screen, process the data, and technically validate the results.

## Methods

### Selecting and validating genes for the KSR1-depletion gene expression signature

To perform a gene-expression high throughput screen (GE-HTS) to identify genes that are functionally similar to KSR1, we developed a gene-expression signature that represents the loss of function of KSR1. One non-targeting shRNA (5′ CAACAAGATGAAGAGCACCAA) and two distinct shRNAs targeting KSR1 (Sequences: 5′ GTGCCAGAAGAGCATGATATT3′ and 5′ GCTCTTCAAGAAAGAGGTGAT 3′) in the pLKO.1-puro vector were individually transfected into HCT116 cells using Lipofectamine 2000, and stable clones were selected using puromycin (4 ug/mL). Over 90% depletion of KSR1 and reduction of anchorage-independent growth were confirmed by Western blot analysis and soft agar colony formation assays, respectively^3^. Total RNA was isolated using TRIzol reagent and RNA quality was assessed using an Agilent2100 BioAnalyzer before reverse transcription, labelling, and hybridization for Affymetrix Human Genome U133A 2.0 gene chips to identify mRNA transcripts that were differentially regulated by the depletion of KSR1. We then assessed the differential expression of genes regulated by stable shRNA-mediated depletion of KSR1 using branched DNA (bDNA) signal amplification (Affymetrix Quantigene 2.0 Multiplex assay). Using the same RNA samples that were used in the microarray analysis, we confirmed that all 12 original candidate genes (KLK10 (Entrez gene ID: 5655), KLK6 (Entrez gene ID: 5653), ARRDC4 (Entrez gene ID: 91947), CA12 (Entrez gene ID: 771), HIG2 (Entrez gene ID: 29923), ADM (Entrez gene ID: 133), LOXL2, ACSL5, NDRG1, ALDOC, BNIP3L, BNIP3) showed differential expression similar to what was demonstrated by microarray analysis.

However, the siRNA screen would not involve stable shRNA-mediated protein depletion so we needed to validate a set of mRNAs that are differentially regulated after transient KSR1 knockdown. We assessed each of the 12 candidate genes for differential expression after transient siRNA-mediated depletion of KSR1 using a siRNA SMARTpool (Dharmacon M-003570-01) at 48 and 72 hours. We have previously shown that the KSR1 SMARTpool provides approximately 90% protein depletion and is similar to stable shRNA-mediated knockdown of KSR1^3^. Although they were upregulated following stable knockdown of KSR1, Kallikrein 6 and 10 (KLK6, KLK10) remained unchanged after transient knockdown of KSR1, suggesting that the altered expression reflected cellular adaptation to KSR1 disruption rather than an acute regulation by KSR1 and these candidate genes were eliminated as reporters of KSR1-dependent activity. One reporter gene, arrestin domain containing 4 (ARRDC4), showed up-regulation at 48 hours, and down-regulation at 72 hours, and was also eliminated as a candidate gene. The other nine candidates demonstrated a KSR1-dependent decrease in expression at both time points. However, six genes (LOXL2, ACSL5, NDRG1, ALDOC, BNIP3L, and BNIP3) demonstrated more consistent down-regulation at 48 and 72 hours. Therefore, these six reporter genes were selected to represent the KSR1-gene expression signature. The six reporter genes were combined with housekeeping genes HPRT and PPIB, which showed no change in expression following loss of KSR1. These invariant housekeeping genes were used to correct for inter-well variability and to normalize the data. A single DNA probe set for the 8 mRNAs was hybridized to each sample then combined with one of eight sets of eight fluorescent Luminex beads allowing the samples to be combined during the signal amplification stage, reducing reagent and labor requirements eight-fold. The eight panels of eight beads/reporters were hybridized to lysates from either control or KSR1 depleted cells, the values were normalized to the geometric mean of PPIB and HPRT, and the ratio of gene expression after KSR1 knockdown compared to the control were assessed for each panel. Minimal variation existed between each panel of beads, confirming the ability to multiplex reagents. Lastly, either control siRNA transfection or siRNA-mediated depletion of KSR1 were performed in 384-well plates, and the resultant lysates were measured using the multiplex Quantigene2.0 assay demonstrating that a KSR1-depletion gene expression signature could be reliably measured in a high-throughput method.

### siRNA screen

This protocol was optimized for HCT116 cells in 384-well format for a final concentration of 50 nM siRNA in a transfection volume of 31 µl containing 2,500 cells/well with 0.05 µl DharmaFECT 4 (DF4) per well. HCT116 cells were purchased from ATCC and maintained in DMEM with 10% fetal bovine serum, L-glutamine, Non-Essential Amino Acids, and Penicillin/Streptomycin and grown at 37 °C in a 5% CO2 incubator. Three million cells were plated into 10-cm culture dishes and allowed to grow for 48 hours before transfection. Each dish will contain approximately 15,000,000 cells at the time of transfection and can be scaled as needed. All work surfaces were cleaned with a detergent cleanser and sterilized with 70% ethanol. 1X Hank’s Buffered Saline Solution (HBSS) was prepared by adding 9.8 g of HBSS powder to 1 L of Sigma HPLC-purified, sterile, RNAse-free, deionized water and filtered through a 0.22 μM filter. No sodium bicarbonate was added. Sets of three Nunc 384-well flat-bottom microtiter tissue culture plates (cat # 164688) were barcode-labelled with proper identifiers. BioRad 384-well polypropylene conical bottom PCR plates (cat # MSP3842) were labelled with proper plate identifiers (one plate/master library screening plate). Master siRNA library plates (384-well format) were removed from −20 °C and allowed to warm to room temperature. Plates were subsequently centrifuged in a Sorvall RC7 tabletop centrifuge (1000 rpm × 3 minutes). In a BCS hood, a 1:106 dilution of DF4/HBSS reagent was prepared to provide enough reagent for all screening plates with extra volume for dilutions, pipetting and a Microdrop liquid dispensing machine void volume of 3 ml. Using a Microdrop dispenser fitted with 11 mm stage (Thermo-Fisher), 15 μl of DF4/HBSS reagent was added to each well of an empty master assay screening plates. Adhesive foil seals were removed from each siRNA containing master screening plate in a BSC hood. 5 uL of 1 μM siRNA control siRNAs (SMARTpool siControl #1: D-001810-01; SMARTpool KSR1: M-003570-01; SMARTpool PPIB: M-004606-00; or SMARTpool PLK1 (Entrez gene ID: 5347): M-003290-01) were added in quintuplicate to each master screening plate by hand. Using a Biomek FX instrument with 384-well tip head, 5 µl of the 1 µM siRNA solution from the master library plate was added to the 15 µl DF4/HBSS mix in its corresponding 384-well master assay screening plate and mixed four times (12 μl aspiration volume). After completion of the transfer, the master screening plate was centrifuged (1000 rpm × 3 minutes) in a Sorvall RT7 tabletop centrifuge and subsequently allowed to sit at room temperature. Simultaneously, the 384-well master library plate was resealed with ABgene PCR heat foil seal and stored in a −80 °C freezer. Triplicate assay screening plates were created with the Biomek FX (384-well tip head) by transferring 6 µl of the siRNA/DF4/HBSS complex from the master assay screening plate to three 384-well Nunc flat-bottom tissue culture screening plates. As the screening plates were being dispensed, HCT116 cells were simultaneously processed to yield 100,000 cells/mL. To do this, medium was removed from three 10-cm dishes and adherent cells washed one time with 5 mL of 1X PBS per dish. Cells were then washed again in 5 mL of 1X PBS with 5 mM EDTA. After 1 minute, the PBS/EDTA solution was removed. Each plate of cells received 1 mL of 0.25% trypsin in PBS and was incubated 5 minutes at 37 °C. Cells were fully detached at this point. Trypsin was neutralized by adding 12 mL of transfection medium (DMEM with 12.5% fetal bovine serum, L-glutamine and Non-Essential Amino Acids) to each dish and the cells were resuspended into a solution of single cells by pipetting up and down. Cells were then passed through a 40 μM screen into a 50 mL Sarstedt conical tube to eliminate clumping. Resuspended cells from each dish were combined, mixed, and counted by hemocytometer. The cell suspension was diluted with additional transfection medium to a final concentration of 100,000 cells/ml in a fresh, sterile, plastic 1 L Erlenmeyer flask and mixed evenly by gently pipetting and swirling. Each 384-well plate required 10 mL of cells at the desired concentration. A typical 10 cm dish yielded at least 10,000,000 cells in 10 mL of media, approximately enough cells for 15 384-well screening plates. Using Multidrop dispenser with 15 mm stage, 25 μL of cell suspension/transfection medium were added to each well of each set of three 384-well transfection plates. Final volume in each well was 31 μL. Cells were not centrifuged before being placed at 37 °C in a 5% CO2 incubator. At 72-hours after transfection cells were lysed using the 96-well head on the Biomek FX by adding to 20 μL of lysis buffer (containing 10 μL of Proteinase K per mL lysis buffer) to each sample and mixed 40 times using 28 μL mixes. The lids were replaced on each plate and placed into a 37 °C incubator. After 60 minutes at 37 °C, each plate was mixed an additional 20 times, sealed with adhesive foil at 175 °C for 4 seconds, and frozen at −80 °C until assayed.

### Gene expression quantitation measured by branched DNA signal amplification (Quantigene 2.0 Multiplex Assay – Panomics/Affymetrix)

The following protocol is based on the processing of two 384-well sample plates after the cells have been lysed (~50 μL/well) and stored at −80 °C. Plates were moved to a 37 °C incubator until entirely thawed. Master mix for one well (30 µL total volume) consisted of 11.8 µL of water, 10 µL of lysis mixture buffer, 0.2 µL of proteinase K, 2.0 µL of blocking reagent, 5 µL of probe mix (Plex Set 11185 contains reagents to measure all 8 genes and the same probe set is used for all 8 bead panels), 1 µL of bead mix (8 different bead panels consisting of 8 uniquely dyed beads for a total of 64 uniquely dyed beads). 1,100 μL of lysis mixture solution (prepared by adding 10 µL of Proteinase K to each mL of lysis mixture) was prepared and warmed in 42 °C water bath to ensure all components were resuspended. 220 µL of blocking solution was thawed from −20 °C. 550 µL of probe mix was thawed from −20 °C and denatured at 95 °C for five minutes. Bead Panels 1–8 were stored at 4 °C and cannot be frozen. The magnetic bead panels were resuspended by vortexing each tube for one min followed by repeated up and down pipetting prior to dispensing. Never sonicate the magnetic beads. Due to the unique nature of the bead panels, 8 separate master mixes were made to accommodate 110 assays to be dispensed to 96 samples. Using two 384-well Greiner brand polypropylene plate plates (cat/ref# 781201), the eight master mixes were pipetted into eight separate 96-well overlapping quadrants i.e. panel 1 into Q1 (upper left hand), panel 2 into Q2 (upper right hand), panel 3 into Q3 (lower left hand), and panel 4 into Q4 (lower right hand) using a micropipette. Panels 5–8 were oriented in the same fashion, respectively, on a 2nd plate. Contents of any well that did not receive a siRNA transfection (columns 1 and 24) were removed and washed once with water then filled with 40 µL water to use in background correction measurements. Using a Biomek FX with the 96-well head attached 40 µL of lysate was added to 30 µL of master mix for a total reaction volume of 70 µL. The lysate solution is viscous and may require the Biomek FX be set to pipet in a larger volume in order to deliver an actual volume of 40 µL. The 384-well plate was heat sealed with ALPS 300 foil at 154 °C for 4 seconds and incubated overnight (18 hours) at 54 °C at max shaking speed in a cytomax2000 shaking incubator. Notes: The incubating shaker could only hold 8 plates at a time. Therefore, the screen was performed in experimental batches/groups (Table 1). The plates were removed from the shaking incubator, the foil seal was removed, and the Biomek FX with the 96-well head was immediately used to move the contents of all 8 quadrants to one quadrant of a new Greiner 384-well plate resting on a magnetic stage. Minimal sample was present on the foil seals so we felt that centrifugation would not impact sample recovery and would potentially reduce bead recovery by pelleting them at the bottom of the well. The beads from 8 samples were condensed into one well of a 384-well plate by adding the samples to the well, allowing the beads to be collected by the magnet for 60 seconds before the supernatant was removed and the next sample was added. Once all the samples had been condensed to one quadrant and the supernatant was removed, 30 µL of pre-amplifier solution (prepared by adding 180 µL of pre-amplifier concentrate to 3.3 mL of label probe diluent warmed to 37 °C) was added to each well, sealed with adhesive foil, and shaken at maximum speed in a cytomax2000 shaking incubator at 50 °C for 60 minutes. 200 mL of wash buffer was prepared by adding 10.0 mL of wash buffer component 2 and 0.6 mL of wash buffer component 1 into 189.4 mL of MilliQ water. After incubation with the pre-amplifier for 60 minutes, the plate was unsealed and placed on the magnetic stage of the Biomek FX and after 60 seconds the contents were removed and the beads were washed three times using 50 µL of wash buffer. 30 µL of amplifier solution (prepared by adding 180 µL of amplifier concentrate to 3.3 mL of label probe diluent warmed to 37 °C) was added to each well, sealed with adhesive foil, and shaken at maximum speed in a cytomax2000 shaking incubator at 50 °C for 60 minutes. After incubation with the amplifier for 60 minutes, the plate is unsealed and placed on the magnetic stage of the Biomek FX and after 60 seconds the contents are removed and the beads were washed three times using 50 µL of wash buffer. 30 µL of biotin label probe solution (prepared by adding 180 µL of amplifier concentrate to 3.3 mL of label probe diluent warmed to 37 °C) was prepared and added to each well, sealed with adhesive foil, and shaken at maximum speed in a cytomax2000 shaking incubator at 50 °C for 60 minutes. After incubation with the biotin label probe for 60 minutes, the plates were unsealed and placed on the magnetic stage of the Biomex FX and after 60 seconds the contents were removed and the beads were washed three times using 50 µL of wash buffer. 30 µL of SAPE reagent was added to each well (prepared by adding 24.75 µL of SAPE concentrate to 3.3 mL of label probe diluent warmed to 37 °C), adhesive sealed, and shaken at max speed on the table top orbital platform shaker for 30 minutes at room temperature cover in tin foil to protect from light. After incubation with the SAPE reagent for 60 minutes, the plate was unsealed and placed on the magnetic stage of the Biomex FX and after 60 seconds the contents were removed and the beads were washed three times using 50 µL of wash buffer. The plate is taken off the magnetic stage and the contents are transferred to a 96 well plate using the Biomex FX with 96-well head using 120 µL of wash buffer. The median fluorescent intensity (MFI) of each of the 64 different beads is determined by measuring at least 50 measurements per bead using a Luminex reader and taking the median value.

**Table 1:** Description of the transfection batches.

Source	Protocol 1	Protocol 2	Protocol 3	Protocol 4	Data
HCT116	siRNA screen	Gene Expression Evaluation via Quantigene 2.0 Assay			PubChem REGID: lewis_rnai_screen_raw
HCT116	siRNA screen	Gene Expression Evaluation via Quantigene 2.0 Assay	Geomean Correction with HPRT and PPIB		PubChem REGID: lewis_rnai_screen_geomean
HCT116	siRNA screen	Gene Expression Evaluation via Quantigene 2.0 Assay	Geomean Correction with HPRT and PPIB	Normalize to siCont wells and Quantify Similarity via Euclidean distance and Pearson correlation	PubChem REGID: lewis_rnai_screen_normalized_results

### Computational processing

A table describing the screen attributes and data that contributed to each of the Data Records is provided (Table 2).

**Table 2:** Description of the screen attributes and data that contributed to each of the Data Records.

Experimental Group/Batch	Plate Numbers
1	1–5, 20–22
2	6–13
3	14–19, 23, 24
4	25–32
5	33–40
6	41–44
7	66, 67

Raw (median fluorescent intensity) MFI measurements from the Luminex instrument representing gene expression for each gene within the 8 gene panel for three technical replicates are available in Pubchem^6^.

#### Data pre-processing

R scripts were used to read and integrate the data. Then the data was reformatted and scrubbed for computational analysis. Once the data was fully integrated and formatted, the values were background subtracted using “blank” wells (40 μl of water and 30 μl of hybridization solution) on a plate-by-plate basis. Then the individual expression values for each gene were divided by the geometric mean (geomean) of housekeeping genes PPIB and HPRT for each well in order to provide expression data of the signature genes corrected for cell number. All processing was completed for each technical replicate individually and on the average of the three technical replicates. Geomean corrected data is available in Pubchem^7^.

#### Normalization

Prior to normalization, negative numbers were set to the probe minimum on each plate. Normalization was completed on a per well basis by dividing each geomean normalized gene expression value by the median value for that signature gene from the negative control (non-targeting siRNA/siCont) wells. After normalization, the data underwent log base 2 transformation.

#### Outliers

Outliers from the repeated wells (*i.e*., control and KSR1 depleted wells) were identified using the grubbs.test algorithm in R, which is based on distance similarity metrics. Minimum number for control wells per plate was set to 6 and for KSR1-depleted wells per batch was set to 20.

#### Calculation of similarity metrics

The positive control, KSR1-depletion target was the average of the KSR1-depleted wells from each experimental batch after outliers were excluded via the grubbs.test algorithm in R as previously described^2–8^. Euclidean distance and Pearson correlation similarity metrics were calculated using the rdist and cor functions in R, respectively. The data normalized to the siCont wells and measured similarity metrics relative to KSR1 depletion are available in Pubchem^8^.

## Data Records

Raw MFI reads for each gene in the 8 gene panel (BNIP3L, NDRG1, ALDOC, LOXL2, BNIP3, ACSL5, HPRT, PPIB) following 14,355 individual gene depletions via pooled siRNA performed in technical triplicate is available at PubChem^6^.

Geomean corrected values based on HPRT and PPIB for each gene in the six reporter gene panel (BNIP3L, NDRG1, ALDOC, LOXL2, BNIP3, ACSL5) following 14,355 individual gene depletions via pooled siRNA performed in technical triplicate is available at PubChem^7^.

Data after normalization to the negative (non-targeting siRNA) controls for each gene and Euclidean distance and Pearson correlation similarity metrics evaluating the similarity relative to the average of the signature from the KSR1 depleted wells in the eight gene panel following 14,355 individual gene depletions via pooled siRNA performed in technical triplicate is available at PubChem^8^.

Samples are defined by the Entrez Gene ID, NCBI Gene Symbol, and NCBI Nucleotide Accession number for the gene that was targeted with siRNA. Additionally, the plate number and plate position are provided.

## Technical Validation

HCT116 cells were purchased from ATCC and maintained in culture by a single researcher. A single lot of FBS (M0017) was purchased for Atlanta Biologics and used for every aspect of this project to ensure consistency.

Probes that have minimal co-variation and change in expression independently are expected to be the most robust reporters of biological activity. To evaluate the covariation of probes, a pairwise comparison between each of the probes was performed on the normalized data and previously reported^4^. BNIP3 and NDRG1 had a correlation value of 0.84 suggesting a moderate level of co-variation, while the remaining pairwise comparisons demonstrated correlations less than 0.7 suggesting the probes move independently.

**Fig. 2:**
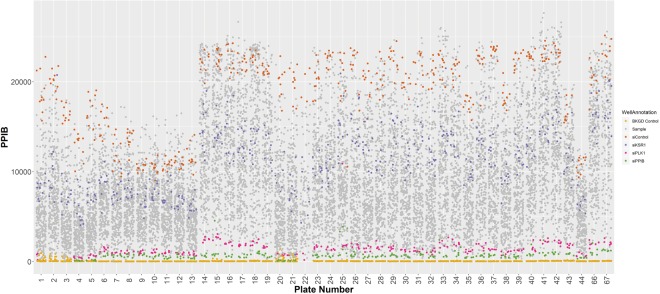
PPIB expression values for control and sample wells to evaluate reproducibility. The PPIB gene expression values for all the controls and siGENOME samples are displayed with respect to that master screening plate where they are located.

Several controls were included to ensure quality data collection including blank wells (wells without cells), a lethal control (siRNA targeting PLK1), a siRNA targeting one of the reporter probes (siRNA PPIB), a positive control (siRNA targeting KSR1), and a non-targeting control (non-targeting siRNA siControl 1). Examining the PPIB expression values relative to the type of control demonstrates several findings (Fig. 2). First, the background control wells are invariably close to zero demonstrating negligible non-specific assay background. Secondly, the PPIB expression values of both the control and sample wells fluctuates most between experimental groups/batches that represent different dates of siRNA transfection (Table 1), and less so between plates that were part of the same transfection batch but assayed separately. This would suggest that limiting the variability between transfections or having a way to normalize between transfection batches is crucial to ensuring high quality data. However, the plates are not randomly distributed as they represent certain function groups within the genome and it might be expected that disruption of the kinases in the genome might have a stronger effect than disrupting a plate full of genomes with unknown function. Thirdly, the transfection efficiency appears very higher as both the lethal control wells and siPPIB wells have consistently low PPIB values with minimal fluctuation. Fourthly, the non-targeting siRNA negative controls consistently have some of the highest PPIB levels and the siKSR1 wells demonstrate intermediate values consistently with the growth inhibitory effects of loss of KSR1.

The potential effect of plate position (*i.e*. plate row and column) was evaluated. PPIB showed a slight trend for increased values on edge rows and HPRT demonstrated an offsetting slight decrease in these rows resulting in minimal differences in the geometric mean. More dramatic effects were seen in gene expression based on column. Columns 1 and 24 were excluded from this analysis because they only contained blank controls. Columns 3 and 22 have lower readings in NDRG1, and ACSL5, as well as a substantially increased range of values for BNIP3 (data not shown); however, this can be attributed to the effect of non-random plating in these columns as these two columns contained five non-targeting negative control wells, five KSR1-depleted wells, five lethal siPLK1 controls, five siPPIB wells and no siGENOME samples. Therefore, no significant effects were demonstrated based on plate position.

Experimental variability between technical replicates was examined and visualized using the raw PPIB gene expression. Replicate and plate consistency were evaluated using PPIB expression (data not shown). There was a high level of correlation between replicates with Pearson correlation values of 0.901, 0.808, and 0.848 between replicates 1 and 2, replicates 1 and 3, and replicates 2 and 3, respectively.

**Fig. 3:**
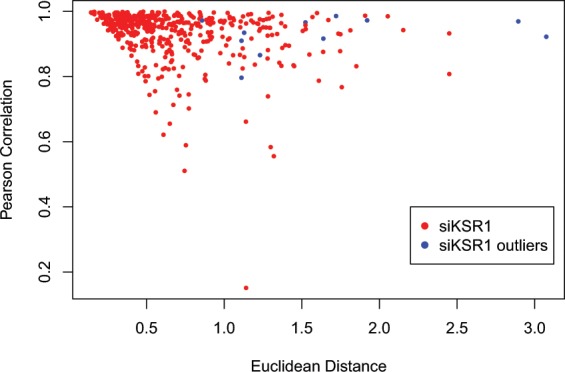
A comparison of all wells with siRNA-mediated knockdown of KSR1. Pearson Correlation and Euclidean Distance of individual wells with siRNA-mediated depletion of KSR1 compared to the average of all the wells with siRNA-mediated depletion of KSR1. Outliers as computer by the Grubbs algorithm are marked as blue dots. Red dots are wells that are not outliers.

**Fig. 4:**
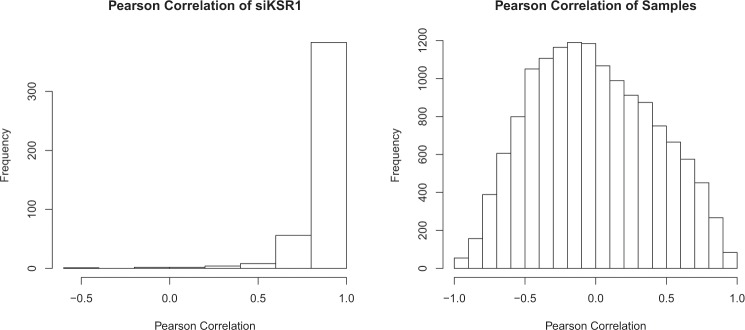
Pearson Correlation of all wells with either siRNA-mediated knockdown of KSR1 compared to its average or siGENOME samples compared to each other. In the left panel, each individual wells with siRNA-mediated knockdown of KSR1 is compared to the average of all wells with siRNA-mediated knockdown of KSR1 and the Pearson Correlation is plotted. In the right panel, all siGENOME samples with the Pearson Correlation to the average of all wells with siRNA-mediated knockdown of KSR1.

Due to the 5 replicates of the siControl and siKSR1 on each plate, we were able to assess the reproducibility and variance associated with biological replicates. In Fig. 3, we show the Pearson Correlation and Euclidean distance of all the individual siKSR1 values compared to the overall average of siKSR1 values and a perfect correlation would be a Pearson correlation of 1.0 and a Euclidean distance approaching zero. Using the Grubbs algorithm, siKSR1 wells colored in blue were removed from the final data set. A pattern to where the outlier wells were located was not identified. One potential cause of unwanted variability may have been from the fact that the siKSR1 wells were hand places on each plate amongst other controls and non-assayed wells. Since the assay was performed by more than one person, it is possible that the handling of some of the control wells was not done properly. However, most of the siKSR1 wells show strong Pearson Correlation and Euclidean Distances and could be used to calculate a robust siKSR1 average and the distance from that average to siGENOME sample wells. The average correlations between individual siKSR1 wells and all siGENOME wells compared to the average of all wells with siRNA-mediated knockdown of KSR1 are shown in Fig. 4, showing a strong correlation between siKSR1 wells and the distribution of correlation score across the genome.

Overall, the findings for the technical validation suggest that the greatest variability is present in the biology, which can be found in the different transfection batches and between individual genetic perturbations. As scientists attempt larger and more complex gene expression based analyses, we suggest standardize technical processing steps, with automation if possible and more importantly, including numerous non-variable mRNAs that can be used to identify and control for variation where it is found.

## Usage Notes

R code for the described pre-processing and computational analysis are included in supplemental Files 1 and 2.

These data can be normalized and similarity can be assessed on an individual plate-basis, on a batch-basis, or across the entire screen. For the KSR1-based analysis, it is recommended that the data be normalized by plate and similarity assessed by batch. However, this can only be done because KSR1 depleted wells are located on each plate. If the similarity between two different genes is examined, the similarity must be assessed across the entire screen. The data was collected in experimental groups or batches due to the limitation of our incubating shaker only being able to hold 8 plates simultaneously. The plate numbers included in each experimental group/batch are listed in Table 1.

The effects of each individual gene depletion on viability can be indirectly assessed by evaluating the raw HPRT and PPIB values^8^ relative to their expression in negative control wells (wells treated with non-targeting siRNA).

To assess the data using a different method of normalization, use the pre-processed data (background subtracted, geomean corrected)^7^.

The normalized data^8^ can be used to assess similarity between any two genes of interest (use a modified form of the provided supplemental file: NormTosiContWellsAndCalcSimilarity.R). It is recommended that the consistency of results be evaluated with respect to the three technical replicates individually as well as an examination of the results from the average of the three technical replicates.

## Supplementary information

Readme

Supplemental File 1

Supplemental File 2

## Data Availability

All of the R code used for the computational analysis described in this report is included within this report as Supplemental Files 1 and 2.

## References

[1] Stegmaier K (2004). Gene expression-based high-throughput screening (GE-HTS) and application to leukemia differentiation. Nat. Genet..

[2] Kortum RL, Lewis RE (2004). The molecular scaffold KSR1 regulates the proliferative and oncogenic potential of cells. Molecular and cellular biology.

[3] Fisher KW (2015). AMPK Promotes Aberrant PGC1beta Expression To Support Human Colon Tumor Cell Survival. Molecular and cellular biology.

[4] Potts MB (2013). Using functional signature ontology (FUSION) to identify mechanisms of action for natural products. Sci. Signal..

[5] McMillan EA (2019). A Genome-wide Functional Signature Ontology Map and Applications to Natural Product Mechanism of Action Discovery. Cell. Chem. Biol..

[6] (2019). PubChem BioAssay.

[7] (2019). PubChem BioAssay.

[8] (2019). PubChem BioAssay.

