# Supplementary material for: High-throughput identification of protein functional similarities using a gene-expression-based siRNA screen
Source: Sci Data. 2020 Jan 21;7:27. doi: 10.1038/s41597-020-0365-2 (PMC6972743; doi:10.1038/s41597-020-0365-2)
Supplement: Supplementary file 1 — Readme [file 41597_2020_365_MOESM1_ESM.docx]

**BKGDSubtAndGeomeanNormInputDataFilesFromADirectory.R**

The purpose of this code is to read and concatenate the data from a single csv file, then background subtract and geomean normalize the data based on the HPRT and PPIB reads to correct for cell number.

**Input:** User selected csv data file. This is designed to be the data from the Results tab of the Data Citation #1 PubChem submission included in this *Scientific Data* Data Descriptor Article saved as a csv file and with the RESULT_DESC and RESULT_TYPE rows deleted so that only a single row containing the column labels (PUBCHEM_RESULT_TAG) remain above the data.

**Output:** A .csv file containing the background subtracted, geomean normalized data. This is equivalent to the results contained within the Results sheet of the Data Citation #2 PubChem submission referenced in this article.

**NormTosiContWellsAndCalcSimilarity.R**

The purpose of this code is to normalize the data to the negative control wells (non-targeting siRNA) and calculate the Euclidean distance and Pearson correlation between all the individual gene depletions and the average of all the KSR1 depleted wells (after outlier removal)

**Input:** User selected csv file that contains the geomean normalized data from all experimental plates. This is designed to be the data from the Results tab of the Data Citation #2 PubChem submission included in this *Scientific Data* Data Descriptor Article saved as a csv file and with the RESULT_DESC and RESULT_TYPE rows deleted so that only a single row containing the column labels (PUBCHEM_RESULT_TAG) remain above the data.

**Output:** A single csv file containing the normalized data and Euclidean distance and Pearson correlation metrics relative to KSR1. This is equivalent to the results contained within the Results sheet of the Data Citation #3 PubChem submission referenced in this article.
